# Retraction Note: Predicting new molecular targets for rhein using network pharmacology

**DOI:** 10.1186/s12918-014-0105-3

**Published:** 2014-09-18

**Authors:** Aihua Zhang, Hui Sun, Bo Yang, Xijun Wang

**Affiliations:** National TCM Key Lab of Serum Pharmacochemistry, Heilongjiang University, of Chinese Medicine, Heping Road 24, Harbin, 150040 China; Key Pharmacometabolomics Platform of Chinese Medicines, Heping Road 24, Harbin, 150040 China

## Retraction

The Editors regretfully retract the article [1] as we believe the peer-review process was compromised and inappropriately influenced by the authors. Following further post-publication peer review the Editors no longer have confidence in the soundness of the findings. We apologise to all affected parties for the inconvenience caused.
